# Preliminary results of posterior contralateral cervical 7 nerve transposition in the treatment of upper limb plegia after a stroke

**DOI:** 10.1002/brb3.1821

**Published:** 2020-09-06

**Authors:** Jingyu Guan, Jun Lin, Xueqing Guan, Qiang Jin, Lei Chen, Qiao Shan, Jianheng Wu, Xiaodong Cai, Doudou Zhang, Wei Tao, Fuyong Chen, Yili Chen, Shaofeng Yang, Youwu Fan, Heming Wu, Han Zhang

**Affiliations:** ^1^ Departments of Neurosurgery General Hospital of Northern Theater Command Shenyang China; ^2^ College of Medicine China Medical University Shenyang China; ^3^ Departments of Anesthesiology General Hospital of Northern Theater Command Shenyang China; ^4^ Departments of Neurosurgery Tianjin Fifth Central Hospital Tianjin China; ^5^ Department of Neurosurgery The Fifth Affiliated Hospital of Zhengzhou University Zhengzhou China; ^6^ Department of Functional Neuro Shenzhen Second People’s Hospital the First Hospital of Shenzhen University Shenzhen China; ^7^ Shenzhen University General Hospital Shenzhen China; ^8^ Department of Neurosurgery Fourth Affiliated Hospital of Medical College of Zhejiang University Yiwu China; ^9^ Department of Neurosurgery Renji Hospital Affiliated to Shanghai Jiaotong University Shanghai China; ^10^ Department of Neurosurgery Nanjing First Hospital Nanjing Medical University Nanjing China; ^11^ Department of Neurosurgery Chengdu Southwest Brain Hospital Chengdu China

**Keywords:** central paralysis, contralateral C7 nerve root, nerve root transfer, posterior spinal route

## Abstract

**Objective:**

This study aimed to explore a shorter and safer contralateral C7 transposition pathway for the treatment of central upper limb paralysis.

**Methods:**

From July 2018 to March 2019, 10 patients with central upper limb paralysis underwent posterior cervical 7 nerve transposition. The age of these patients ranged within 31–58 years old (average: 44 years old). These patients comprised of eight male patients and two female patients. Nine patients had cerebral hemorrhage, and one patient had a cerebral infarction. Furthermore, nine patients presented with spastic paralysis of the upper limbs and one patient presented with nonspastic paralysis. The duration of plegia before the operation ranged from 6 to 60 months (average: 26 months). The surgical procedure included transposition of the contralateral cervical 7 nerve root via a posterior vertebral approach under general anesthesia, and the distal part of the contralateral cervical 7 nerve was anastomosed with the proximal part of the ipsilateral cervical 7 nerve.

**Results:**

The length of the contralateral cervical 7 nerve was 5.16 ± 0.21 cm, which was directly anastomosed with the ipsilateral cervical 7 nerve. Neither case needed nerve transplantation. Most patients had temporary numbness in their healthy fingers, which all disappeared within three months. Up to now, the follow‐up results are as follows: The spasticity of the affected upper limbs in five patients is lower than that before the operation, the pain and temperature sensation of the affected upper limbs in six patients are better than before the operation.

**Conclusion:**

The distance of nerve transposition can be shortened by a posterior vertebral approach operation, where the contralateral C7 nerve can be anastomosed directly with the ipsilateral C7 nerve which may be effective for nerve regeneration and functional recovery. However, this conclusion still needs further research and verification.

## Graphical abstract

This study aimed to explore a shorter and safer contralateral C7 transposition pathway for the treatment of central upper limb paralysis. The distance of nerve transposition can be shortened by a posterior vertebral approach operation, where the contralateral C7 nerve can be anastomosed directly with the ipsilateral C7 nerve. In this approach, nerve transplantation is not needed and it is conducive to nerve regeneration and functional recovery.

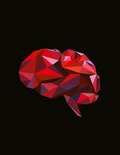

## 1 INTRODUCTION

A stroke can cause many serious sequelae, one of which is central upper limb plegia. Plegia results in the patient being unable to take care of himself/herself and has a great impact on the patient's life (Zheng, Hua, & Feng, 2018). A previous study revealed that restoring the ipsilateral motor cortex function on the side of the paralyzed limbs is the physiological basis for the functional recovery of the paralyzed limbs. In the past, activating the ipsilateral motor cortex function was performed during rehabilitation therapy; however, the individual difference of this therapy is great and the effect is not satisfactory (Hua et al., 2015).

Professor XuWendong first applied cervical 7 nerve transposition to treat central upper limb plegia and achieved remarkable results allowing for a better therapy procedure that minimizes the technical disadvantages of rehabilitation of the central plegia. The surgical approach includes the commonly used anterior vertebral approach (Wang et al., 2012, 2013; Xu, Hua, Zheng, Xu, & Gu, 2011). The disadvantage of the anterior vertebral approach is that the length of the contralateral cervical 7 nerve is insufficient in some patients and it is difficult to achieve a one‐stop anastomosis. In this case, nerve transplantation is needed, which affects the recovery effect. In addition, there are complications such as aortic injury, esophageal basket, and upper limb pain during swallowing (Xu et al., 2011).

Therefore, taking into account the disadvantages of the anterior vertebral approach, the investigators explored the feasibility of the posterior vertebral approach. In July 2018, lateral cervical 7 nerve transposition via the posterior vertebral approach was first performed for the treatment of central upper limb plegia (Xu et al., 2011). Subsequently, the investigators successively completed 10 operations. All the 10 patients achieved a one‐stop nerve anastomosis without nerve transplantation. Preliminary follow‐up results after the operations are reported as follows.

## 2 PATIENTS AND METHOD

### 2.1 General information of the patients

From July 2018 to March 2019, 10 patients with central upper limb paralysis underwent posterior cervical 7 nerve root transposition in our department. The age of these patients ranged within 31–58 years old (average: 44 years old). These patients comprised of eight male patients and two female patients. Nine patients suffered from a cerebral hemorrhage, and one patient had a cerebral infarction. Nine patients had secondary spastic paralysis of the upper limbs, and one patient had nonspastic paralysis. The duration of plegia before the operation ranged from 6 to 60 months (average: 26 months). This study was conducted with approval from the Ethics Committee of General Hospital of Northern Theater Command. This study was conducted in accordance with the declaration of Helsinki. Written informed consent was obtained from all participants. All patients received follow‐ups until March 2019.

### 2.2 Inclusion and exclusion criteria

Inclusion criteria were as follows: (a) Patients who were diagnosed as central upper limb paralysis underwent posterior cervical 7 nerve root transposition; (b) age was older than 18 years old; and (c) patients who have signed informed consent. Exclusion criteria were as follows: (a) patients who had severe infection; (b) patients who had severe heart, liver, or kidney dysfunction; (c) patients who had severe coagulation dysfunction; and (d) patients whose data were incomplete.

### 2.3 Operative approach

**Figure 1:**
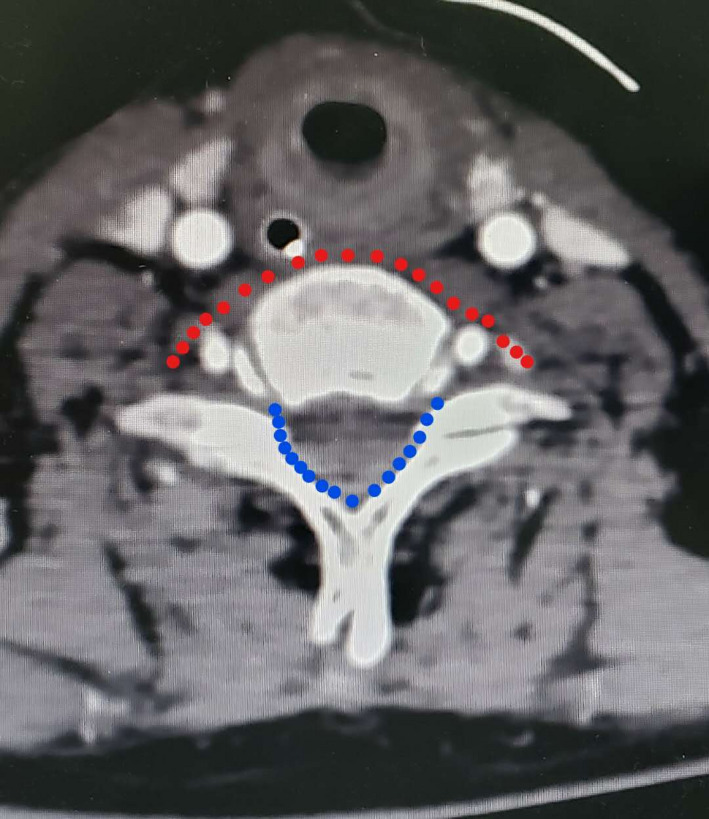
The red arc is the nerve pathway in the anterior vertebral approach, and the blue arc is the nerve pathway in the posterior vertebral approach (It can be seen that the pathway in the posterior approach is significantly shorter than that in the anterior approach)

A affected supraclavicular transverse incision combined with the posterior cervical median incision was adopted in this study. Patients were first placed in the supine position. The affected cervical 7 nerve was determined and separated through the affected supraclavicular transverse incision, the proximal end was separated into the foramen intervertebralis, and the distal end was cut off at the commissural trunk. After completing the measurement, the nerve was marked and the supraclavicular incision was sutured. Then, the patients were placed in the prone position. The middle site of the posterior cervical spinous processes 6, 7 was taken as the center of the straight incision, bilateral foramens of cervical 6, 7 were opened, and the cut affected cervical 7 nerve was pulled out from the intervertebral foramen. The cervical 6 and 7 spinous processes were drilled near the lamina to form a channel of approximately 1 cm, and the lamina was ground open, a groove of approximately 1 cm was formed in the ligamentum flavum, and the distal part of the left cervical 7 nerve passed through the foramen and left lying above the ligamentum flavum in the groove. The cervical 7 nerve was cut in the middle of the intervertebral foramen of the affected side and anastomosed with the affected cervical 7 nerve in a tension‐free manner (Figure 1). Bilateral superior and inferior articular processes were fixed, and the incision was sutured layer by layer. After the operation, the patients’ necks were fixed with a cervical bracket and the motion of the right upper limb was properly restrained. In our study, the articular processes under the grinding and grinding were connected and fixed in situ by connecting plates. There were no implants. Postoperative patients received rehabilitation. As the site of nerve repair moved from proximal to distal, the rehabilitation frontal key muscle group was different. Two observers who participated in the postoperative follow‐up of 10 patients knew that the patients had undergone surgery, but not included the specific data.

### 2.4 Detection indicators

The detection indicators included the length of the affected cervical 7 nerve, the complications of the operation, the symptoms after the operation, and the symptoms during the follow‐up sessions.

## 3 RESULTS

### 3.1 Overall results

The length of the contralateral cervical 7 nerve was 5.16 ± 0.21 cm in all 10 patients. In all patients, the unaffected cervical 7 nerve was directly anastomosed with the ipsilateral cervical 7 nerve and no patient needed additional nerve transplantation. No complications occurred in all operations. The esophagus, great vessels, pleura, or thoracic duct was not damaged during and after the operation.

Most patients had temporary numbness in their healthy fingers, which all disappeared within three months. In the follow‐up sessions in March 2019, the spasticity of the affected upper limbs in five patients was lower than that before the operation and the pain and temperature sensation of the affected upper limbs in six patients were better than before the operation. The rankin score, Fugl‐Meyer scale, Barthel index, and Ashworth were listed in Table 1.

**Table 1:** The rankin score, Barthel index, and Ashworth

Patient	Age	Sex	Fugl‐Meyer scale	Rankin score		Barthel index		Ashworth	
				Before surgery	After surgery	Before surgery	After surgery	Before surgery	After surgery
1	58	Female	26	3	3	30	40	Shoulder:0; elbow:0; hand:0; wrist:0	Shoulder:0; elbow:0; hand:0; wrist:0
2	36	Male	30	3	3	60	75	Shoulder:I; elbow:II; hand:III; wrist:III	Shoulder:I; elbow:I; hand:I; wrist:I
3	44	Male	36	3	3	60	80	Shoulder:I; elbow:II; hand:III; wrist:III	Shoulder:I; elbow:I; hand:I; wrist:I
4	37	Male	34	3	2	60	75	Shoulder:I; elbow:II; hand:III; wrist:III	Shoulder:I; elbow:II; hand:I; wrist:I
5	36	Male	32	3	3	60	80	Shoulder:I; elbow:II; hand:II; wrist:III	Shoulder:I; elbow:I; hand:II; wrist:I
6	49	Female	58	3	2	40	75	Shoulder:I; elbow:II; hand:III; wrist:III	Shoulder:I; elbow:I; hand:I; wrist:I
7	60	Male	36	3	3	65	75	Shoulder:I; elbow:II; hand:II; wrist:III	Shoulder:I; elbow:I; hand:II; wrist:II
8	48	Male	28	3	3	60	75	Shoulder:I; elbow:II; hand:III; wrist:III	Shoulder:I; elbow:I; hand:II; wrist:II
9	31	Male	28	3	3	55	75	Shoulder:I; elbow:II; hand:III; wrist:III	Shoulder:I; elbow:I; hand:II; wrist:II
10	41	Male	26	3	3	30	50	Shoulder:I; elbow:II; hand:III; wrist:III	Shoulder:I; elbow:I; hand:II; wrist:II

### 3.2 Typical cases

#### 3.2.1 Patient information

**Figure 2:**
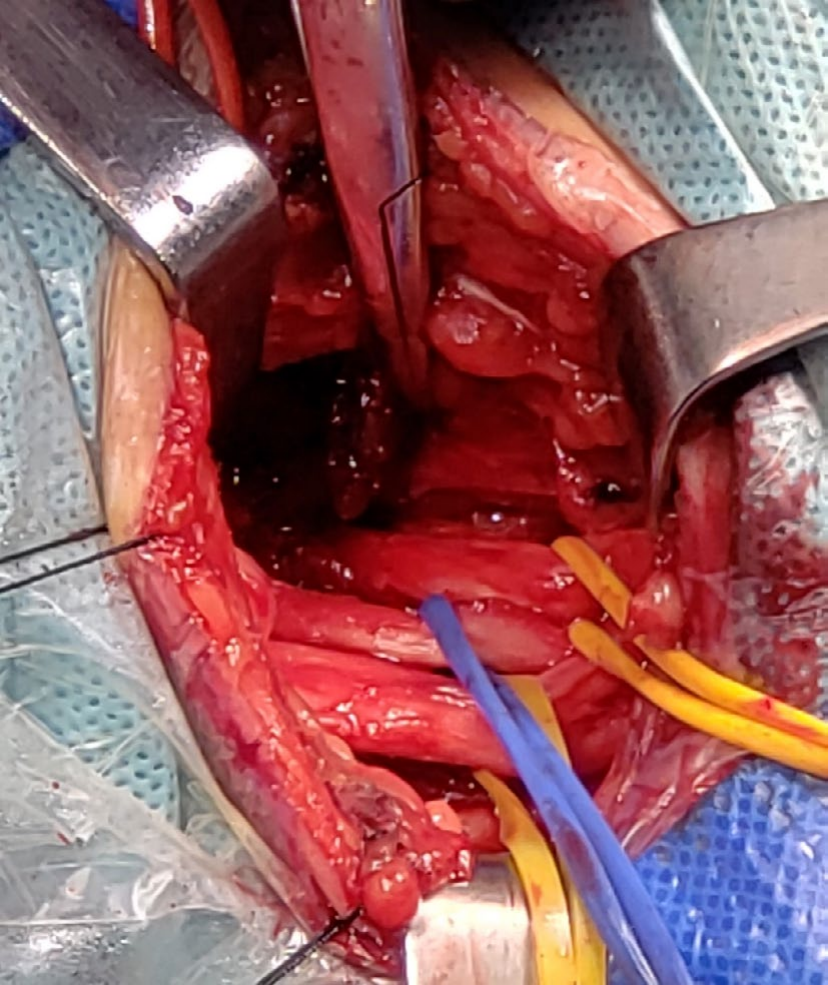
Exposing the contralateral brachial plexus

**Figure 3:**
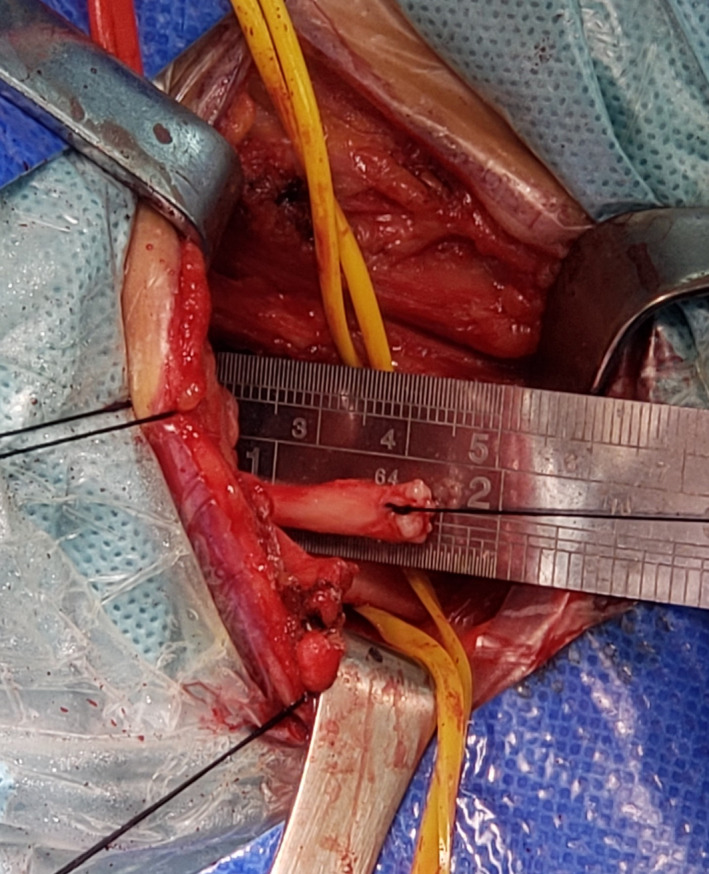
Cutting and measuring the contralateral cervical 7 nerve

**Figure 4:**
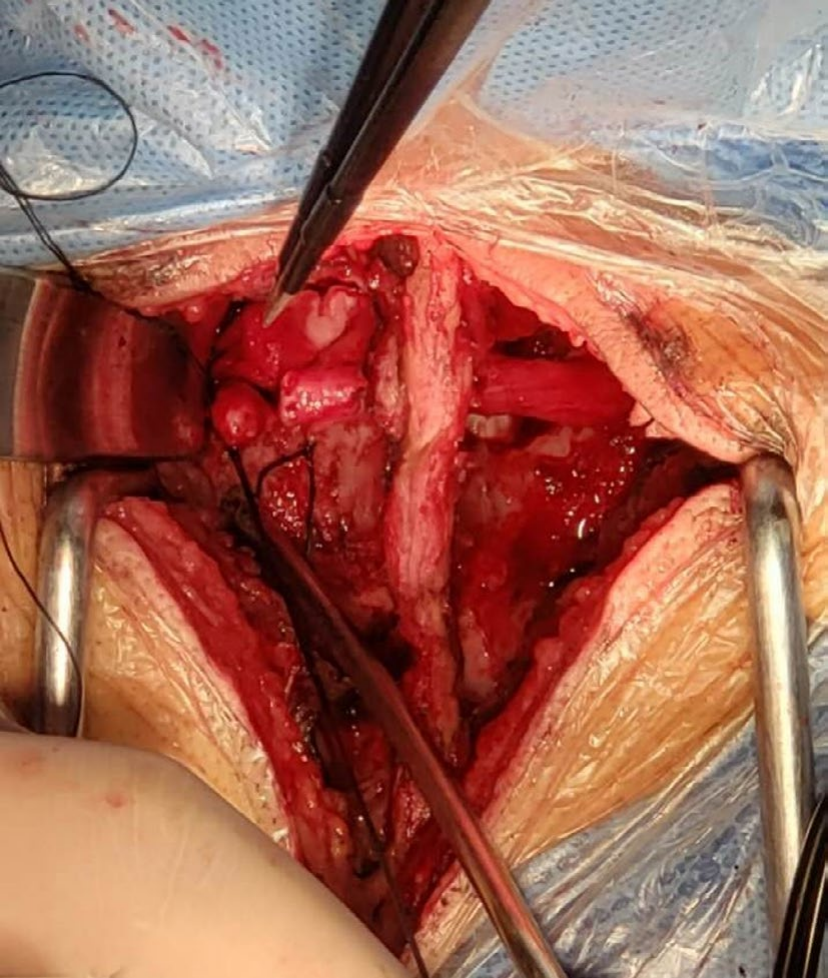
In the posterior approach, the contralateral cervical 7 nerve is pulled out from the spinous process space above the ligamentum flavum and is anastomosed with the proximal end of the affected side in a tension‐free manner

**Figure 5:**
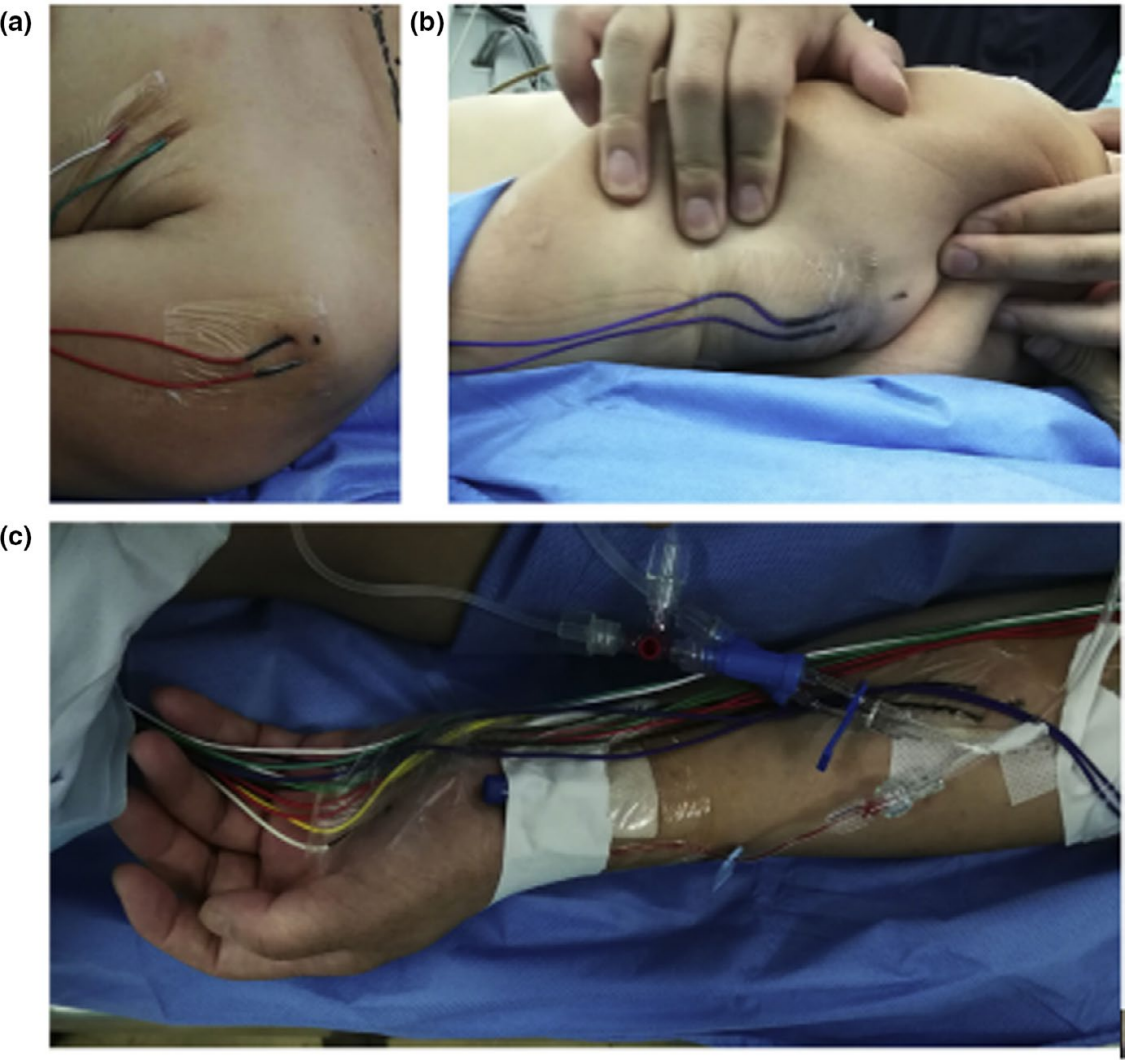
Intraoperative electrophysiological monitoring. (a) The left deltoid muscle and the biceps muscle were monitored for the C5 and C6 nerves. (b) The triceps long head was monitored for the C7 nerve. (c) The flexor carpi ulnaris muscle and muscle of thenar were monitored for the C8 and chest

**Figure 6:**
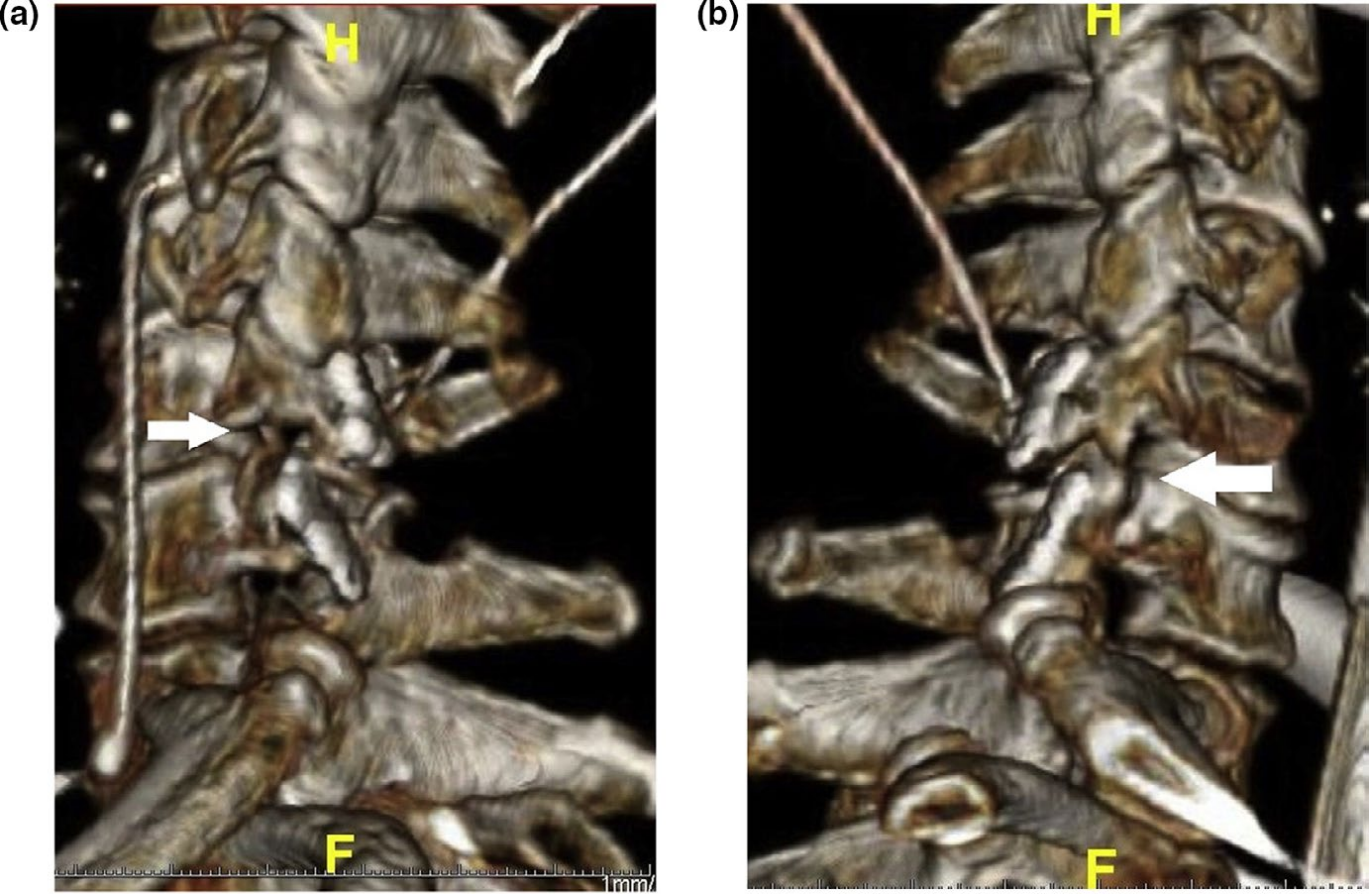
Three‐dimensional reconstruction of the cervical spine after operation. (a) The left upper and lower articular processes are fixed (arrows). (b) The right upper and lower articular processes are fixed (arrows)

Case 5 was a 36‐year‐old female patient. One year ago, the right limb was paralyzed because of the patient experiencing a cerebral hemorrhage in the left basal ganglia. A physical examination showed that the muscle strength of the right lower extremity was grade 4. The patient needed to walk with the help of others. The right upper limb had high spasticity, and the patient's elbow was flexed. The pain and temperature sensations decreased. Proximal muscle strength was level 3, distal muscle strength was level 2, and the Fugl‐Meyer score was 16 points. Moreover, an EMG showed that the signals to the right upper limb were weakened. The details of surgery were showed in Figures 2, 3, 4, 5, 6.

#### 3.2.2 Follow‐up results

At three months after the operation, the spasticity of the right upper limb decreased significantly, the elbow joints could be placed in a naturally vertical manner, and the patient's pain and temperature sensations were better than before the operation. The patient no longer needed support when walking, and the Fugl‐Meyer score was 26 points.

## 4 DISCUSSION

The outcomes of this study presented that the length of the contralateral cervical 7 nerve was directly anastomosed with the ipsilateral cervical 7 nerve. Neither case needed nerve transplantation. Most patients had temporary numbness in their healthy fingers, which all disappeared within three months. The spasticity of the affected upper limbs in five patients was lower than that before the operation, and the pain and temperature sensation of the affected upper limbs in six patients are better than before the operation. Therefore, the distance of nerve transposition can be shortened by a posterior vertebral approach operation, where the contralateral C7 nerve can be anastomosed directly with the ipsilateral C7 nerve which may be effective for nerve regeneration and functional recovery. However, this conclusion still needs further research and verification.

Brachial plexus is composed of C5 nerve, C6 nerve, C7 nerve, C8 nerve, and 1 cervical nerve. There were 20% C7 nerve components in C5 nerve, C6 nerve, C8 nerve, and pectoral 1 nerve, respectively. Therefore, the function of all brachial plexus nerves can be improved with the nerve repair after translocation of contralateral C7 nerve. Previous studies have reported the hemiplegia cases which required nerve grafting for C7 ‐ C7 transfer via prespinal route (Li et al., 2015; Wang, Hu, & Wang, 2003). Academician GuYudong (Guan, Lin, Guan, & Qiang, 2019) discovered for the first time that the contralateral cervical 7 nerve could provide a powerful source of the dynamic nerve without obvious damage to the contralateral function and first innovated the contralateral cervical 7 nerve transposition through anterior cervical subcutaneous approach, which has become a classic surgical procedure of contralateral nerve root transposition. However, in this approach, the nerve needs to be bridged long. Mcguiness et al. (Gu et al., 1992) reported for the first time and clinically applied the anterior vertebral approach, which shortened the length of the bridged nerve graft. Professor Xu Lei et al. (Mcguiness & Kay, 2002) further improved the anterior vertebral approach, where instead, the nerve passes through the pharynx and the anterior trapezius muscle is cut off, which further shortens the nerve pathway and allows many patients to achieve direct anastomosis; therefore, the required pathway is shorter. However, there are still a few patients who need a bridging nerve because of the insufficient length of the contralateral cervical 7 nerve (Xu et al., 2008).

In order to overcome this disadvantage of the anterior vertebral approach, the investigators first performed lateral cervical 7 nerve transposition via the posterior vertebral approach for the treatment of central upper limb plegia (Leblebicioglu et al., 2016). Since then, another 10 cases have been operated using this procedure. The length of the contralateral cervical 7 nerve separated during the operation was 5.16 ± 0.21 cm. Subsequently, when we opened the contralateral facets and continued to separate the nerves, we could increase the length by approximately 1 cm and the distance of the nerve pathway was approximately 4 cm. In this way, tension‐free direct anastomosis was achieved in all patients and neither patient needed nerve bridging.

After the operation, the preliminary follow‐up results revealed that the frequency of spasmodic episodes was reduced and temperature sensation had improved in 7 patients. However, further follow‐up sessions to determine the improvement of hand motor function is needed because of the short time between the operation and the follow‐up sessions.

In addition, this surgical procedure also needs the patient to change their body position to increase the operation time. In order to combine the anterior and posterior approaches and resolve the problem of large surgical trauma, the investigators are also exploring the feasibility of completing one operation in the lateral position and the feasibility to further reduce trauma and completely avoid anterior incision trauma by combining the application of endoscopy (Dhawan, Kennedy, Rizk, & Ozbolat, 2019; Lipskas, Deep, & Yao, 2019; Wei, Guo, Ji, Zhang, & Liang 2017).

In addition, due to the shortening of the articular process after grinding, only part of the articular process can be preserved after the reduction. Although it does not affect cervical stability, however, from the perspective of complete restoration of anatomy, if 3D printing of the articular processes (Serrão, de Araújo, Couto Neto, Harley Santos Botelho, & Carpi, 2017) can be used, the restoration effect will be better. Furthermore, studying the sheath wrapping around the nerve anastomosis and promoting the nerve regeneration speed is also the direction of further research (Hsu, Chang, & Yen, 2017).

Limitations. There were several limitations in this study. Firstly, this study was only single‐center trial and the sample size was limited. Secondly, the duration of upper limb paralysis before the surgery was very variable among patients, from 6 to 60 months, which may influence the results to some extent. Thirdly, these five patients after surgery improved 10 points in the total score of the Fugl‐Meyer Assessment scale, However, a minimal clinically important difference score for the upper extremity motor recovery was defined as 9 to 10 on the FMA‐UE. Therefore, the sensorimotor impairment still needs further research.

## 5 CONCLUSIONS

For upper limb plegia after a stroke, contralateral cervical 7 nerve transposition through the posterior vertebral approach can shorten the distance of nerve transposition and it allows the contralateral C7 nerve to be anastomosed directly with the ipsilateral C7 nerve without needing nerve transplantation and is conducive to nerve regeneration and functional recovery. This method is a safe and effective treatment for central upper limb paralysis after a stroke.

## CONFLICT OF INTEREST

The authors declare that they have no competing interests.

## Data Availability

We declared that materials described in the manuscript, including all relevant raw data, will be freely available to any scientist wishing to use them for noncommercial purposes, without breaching participant confidentiality.

## References

[brb31821-bib-0001] Dhawan, A. , Kennedy, P. M. , Rizk, E. B. , & Ozbolat, I. T. (2019). Three‐dimensional bioprinting for bone and cartilage restoration in orthopaedic surgery. Journal of the American Academy of Orthopaedic Surgeons, 27, e215–e226. 10.5435/JAAOS-D-17-00632 30371527

[brb31821-bib-0002] Gu, Y. D. , Zhang, G. M. , Chen, D. S. , Yan, J. G. , Cheng, X. M. , & Chen, L. (1992). Seventh cervical nerve root transfer from the contralateral healthy side for treatment of brachial plexus root avulsion. Journal of Hand Surgery, 17, 518–521. 10.1016/S0266-7681(05)80235-9 1479244

[brb31821-bib-0003] Guan, J. , Lin, J. , Guan, X. , & Qiang, J. (2019). Treatment of central paralysis of the upper extremity using contralateral C7 nerve transfer via the posterior spinal route ‐ A case report. World Neurosurgery, 125, 228–233. 30738934 10.1016/j.wneu.2019.01.181

[brb31821-bib-0004] Hsu, S. H. , Chang, W. C. , & Yen, C. T. (2017). Novel flexible nerve conduits made of water‐based biodegradable polyurethane for peripheral nerve regeneration. Journal of Biomedical Materials Research Part A, 105, 1383–1392. 28152586 10.1002/jbm.a.36022

[brb31821-bib-0005] Hua, X. Y. , Qiu, Y. Q. , Li, T. , Zheng, M. X. , Shen, Y. D. , Jiang, S. , … Xu, W. D. (2015). Contralateral peripheral neurotization for hemiplegic upper extremity after central neurologic injury. Neurosurgery, 76, 187–195. 10.1227/NEU.0000000000000590 25549193

[brb31821-bib-0006] Leblebicioglu, G. , Ayhan, C. , Firat, T. , Uzumcugil, A. , Yorubulut, M. , & Doral, M. N. (2016). Recovery of upper extremity function following endoscopically assisted contralateral C7 transfer for obstetrical brachial plexus injury. The Journal of Hand Surgery, 46, 863–874. 10.1177/1753193416638999 26988920

[brb31821-bib-0007] Li, W. , Wang, S. , Zhao, J. , Rahman, M. F. , Li, Y. , Li, P. , & Xue, Y. (2015). Complications of contralateral C‐7 transfer through the modified prespinal route for repairing brachial plexus root avulsion injury: A retrospective study of 425 patients. Journal of Neurosurgery, 122(6), 1421–1428. 10.3171/2014.10.JNS131574 25495742

[brb31821-bib-0008] Lipskas, J. , Deep, K. , & Yao, W. (2019). Robotic‐assisted 3D bio‐printing for repairing bone and cartilage defects through a minimally invasive approach. Scientific Reports, 9, 3746 10.1038/s41598-019-38972-2 30842477 PMC6403301

[brb31821-bib-0009] Mcguiness, C. N. S. , & Kay, S. P. (2002). The prespinal route in contralateral C7 nerve root transfer for brachial plexus avulsion injuries. Journal of Hand Surgery, 27, 159–160. 10.1054/JHSB.2001.0665 12027492

[brb31821-bib-0010] Serrão, C. , de Araújo, G. , Couto Neto, B. , Harley Santos Botelho, R. , & Carpi, M. M. (2017). Clinical evaluation after peripheral nerve repair with caprolactone neurotube. Hand (N Y), 12, 168–174. 10.1177/1558944716643277 28344529 PMC5349409

[brb31821-bib-0011] Wang, S. F. , Hu, Q. , & Wang, H. H. (2003). The anatomical and clinical study of contralateral C_7 transfer through the prespinal route. Chinese Journal of Hand Surgery, 19(2), 69–71.

[brb31821-bib-0012] Wang, S. F. , Li, P. C. , Xue, Y. H. , Yiu, H. W. , Li, Y. C. , & Wang, H. H. (2013). Contralateral C7 nerve transfer with direct coaptation to restore lower trunk function after traumatic brachial plexus avulsion. Journal of Bone and Joint Surgery. American Volume, 95, 821–827. 10.2106/JBJS.L.00039 23636189

[brb31821-bib-0013] Wang, S. , Yiu, H. W. , Li, P. , Li, Y. , Wang, H. , & Pan, Y. (2012). Contralateral C7 nerve root transfer to neurotize the upper trunk via a modified prespinal route in repair of brachial plexus avulsion injury. Microsurgery., 32, 183–188. 10.1002/micr.20963 22002908

[brb31821-bib-0014] Wei, R. , Guo, W. , Ji, T. , Zhang, Y. , & Liang, H. (2017). One‐step reconstruction with a 3D‐printed, custom‐made prosthesis after total enbloc sacrectomy: A technical note. European Spine Journal, 26, 1902–1909. 10.1007/s00586-016-4871-z 27844229

[brb31821-bib-0015] Xu, L. , Gu, Y. , Xu, J. , Lin, S. , Chen, L. , & Lu, J. (2008). Contralateral C7 transfer via the prespinal and retropharyngeal route to repair brachial plexus root avulsion: A preliminary report. Neurosurgery, 63, 553–558. 10.1227/01.NEU.0000324729.03588.BA 18812967

[brb31821-bib-0016] Xu, W. D. , Hua, X. Y. , Zheng, M. X. , Xu, J. G. , & Gu, Y. D. (2011). Contralateral C7 nerve root transfer in treatment of cerebral palsy in a child: Case report. Microsurgery., 31, 404–408. 10.1002/micr.20877 21503970

[brb31821-bib-0017] Zheng, M. X. , Hua, X. Y. , Feng, J. T. , Li, T. , Lu, Y.‐C. , Shen, Y.‐D. , … Xu, W.‐D. (2018). Trial of contralateral seventh cervical nerve transfer for spastic arm paralysis. New England Journal of Medicine, 378, 22–34. 10.1056/NEJMoa1615208 29262271

